# Research Advances in the Mechanisms of Hyperuricemia-Induced Renal Injury

**DOI:** 10.1155/2020/5817348

**Published:** 2020-06-26

**Authors:** Hong-yong Su, Chen Yang, Dong Liang, Hua-feng Liu

**Affiliations:** Key Laboratory of Prevention and Management of Chronic Kidney Disease of Zhanjiang City, Institute of Nephrology, Affiliated Hospital of Guangdong Medical University, Zhanjiang, Guangdong 524001, China

## Abstract

Uric acid is the end product of purine metabolism in humans, and its excessive accumulation leads to hyperuricemia and urate crystal deposition in tissues including joints and kidneys. Hyperuricemia is considered an independent risk factor for cardiovascular and renal diseases. Although the symptoms of hyperuricemia-induced renal injury have long been known, the pathophysiological molecular mechanisms are not completely understood. In this review, we focus on the research advances in the mechanisms of hyperuricemia-caused renal injury, primarily on oxidative stress, endothelial dysfunction, renal fibrosis, and inflammation. Furthermore, we discuss the progress in hyperuricemia management.

## 1. Introduction

Uric acid (2,6,8-trioxypurine, molecular formula C_5_H_4_N_4_O_3_; UA), the final metabolite of endogenous and exogenous purine, is generated in the liver [1]. Owing to the evolutionary loss of urate oxidase, UA cannot be catabolized to allantoin in humans and primates, while most of other animals can catabolize UA [1]. As a major antioxidant [2], UA is beneficial to remove superoxide and oxygen free radicals in primates, which is unable to self-generate vitamin C [3, 4]. Hyperuricemia is clinically defined as the serum UA level ≥ 7 mg/dL in men and postmenopausal women and ≥6 mg/dL in premenopausal women, causing various diseases, such as gout and urinary stones [3–5]. An elevated UA level is also tightly associated with diabetes and cardiovascular and kidney diseases [6]. Recently, because of the changes in lifestyle and the increasing population of older people, the incidence of hyperuricemia in China has risen from merely 1.4% in the early 1980s to 10% in the early 21st century [7]. In fact, the morbidity from hyperuricemia has risen up to 20% in some coastal areas and developed cities in China, nearly reaching the level of developed countries [8, 9].

Numerous studies have shown that hyperuricemia is closely associated with kidney diseases [2, 10–13]. In a 6-year cohort study of 10,677 Chinese individuals with a normal estimated glomerular filtration rate (eGFR) and without proteinuria, a higher UA level was found to contribute to the onset of kidney disease and a rapid decline of eGFR [14]. A long-term follow-up cohort study of 13,338 volunteers with normal kidney function in two communities showed a significant relationship between the baseline UA level and the risk of kidney disease, with the risk of developing kidney disease rising by 7% to 11% per 1 mg/dL serum UA [15]. Another cohort study, which lasted for more than 25 years and enrolled 177,570 patients, showed an independent association between high UA levels and end-stage renal diseases (ESRDs) [16]. Hyperuricemia is now considered an independent risk factor for the occurrence and development of diabetic nephropathy (DN) [17, 18], acute kidney injury (AKI) [2, 12, 13], chronic kidney disease (CKD) [10, 11], and ESRD [16]. However, inconsistent results had been reported regarding the role of UA in the progression of CKD and there were insufficient evidences to suggest lowering UA therapy to prevent the progression of CKD [19–23]. Thus, the causal relationship between hyperuricemia and CKD remains controversial, and the pathophysiological mechanisms of hyperuricemia-induced renal injury are not entirely clear. In this review, we attempt to elucidate the recent advances in the mechanisms of hyperuricemia-induced renal injury.

## 2. Pathogenesis of Hyperuricemia

The cause of hyperuricemia is the imbalance between UA production and excretion. Purines are mainly degraded by xanthine oxidase (XO) in the liver, and targeting this enzyme, e.g., with allopurinol or febuxostat, is an effective therapeutic method for lowering serum UA levels [24]. A high-purine diet, increased purine metabolism, and excessive alcohol consumption contribute to the increased production of UA. Tumor lysis syndrome, in which a large number of cells are damaged, and the metabolism of nucleic acids is promoted, leads to an increase in UA production [25]. Other rare causes inducing acute hyperuricemia include seizures, rhabdomyolysis, and excessive exercise.

Most of UA is excreted by the kidneys (65–75%) and intestines (25–35%). The renal handling of UA consists of glomerular filtration, tubular reabsorption, tubular secretion, and reabsorption after secretion. A decrease in UA excretion and an increase in UA reabsorption cause hyperuricemia. UA transporters are required for the renal handling of UA and can be roughly divided into reabsorption-related and secretion-related proteins. Reabsorption-related proteins mainly include urate anion transporter 1 (URAT1), organic anion transporter 4 (OAT4), and glucose transporter 9 (GLUT9), while secretion-related transporters mainly consist of OAT1, OAT3, multidrug resistance protein 4 (MRP4/ABCC4), and breast cancer resistance protein (BCRP/ABCG2) [26]. For example, the function of URAT1 is to reabsorb UA at the apical membrane of proximal tubule epithelial cells (TECs) [27, 28]. GLUT9 acts as a transporter that reabsorbs both UA and glucose into tubular cells [26]. ABCG2, which was first found to be involved in the development of multidrug resistance in cancer cells, also takes part in the secretion of UA from proximal TECs through an ion pump [29]. Genetic defects or mutations in secretion-related transporters also contribute to hyperuricemia. In addition, some drugs, such as cyclosporine and diuretics, cause hyperuricemia by decreasing renal urate clearance [30].

## 3. Mechanisms of Hyperuricemia-Induced Renal Injury

### 3.1. Monosodium Urate (MSU) Crystal Deposition-Induced Renal Damage

UA has the characteristic of a weak organic acid, and most of it is ionized to MSU crystal at pH 7.4 and a temperature of 37°C [31, 32]. A solubility study showed that serum was supersaturated for MSU crystal when the concentration of UA exceeded 6.5 mg/dL [31]. As a consequence, UA and urate crystals may deposit in the joints, kidneys, and other tissues, inducing tissue damage. A reduced expression of BCRP/ABCG2 in TECs induced by hyperuricemia may promote MSU crystal deposition in TECs and the renal interstitium, resulting in substantial renal damage [33, 34]. Under long-term low pH and high UA concentration conditions in crude urine, urate crystal deposition in the renal tubular lumen and ureters contributes to cast formation and obstructive nephropathy [35–37]. Upon obstruction, a series of complications occur, such as local damage, infection, bleeding, and hydronephrosis [38]. In particular, insulin resistance leads to impaired UA excretion at a low urinary pH, contributing to the formation of urate stones [39].

### 3.2. Hyperuricemia-Induced Oxidative Stress

Once transported into the cell, UA becomes a prooxidant, which increases the production of reactive oxygen species (ROS), including the superoxide anion (O_2_^−^), H_2_O_2_, and 8-isoprostane [40, 41]. Many studies have shown that hyperuricemia-induced oxidative stress affects multiple organs and systems, including the kidneys [42–44]. Pathologically, hyperuricemia-associated oxidative stress gives rise to DNA damage, oxidation and inactivation of enzymes, inflammatory cytokine production, and cell apoptosis [45].

Mitochondria are the center of intracellular energy metabolism and the main site of oxidative phosphorylation, in which ROS are produced by the transfer of electrons from electron transport chain complexes to O_2_. It has been reported that long-term hyperuricemia could induce renal mitochondria dysfunction associated with renal cortex oxidative stress and tubular damage in rats [46]. Hyperuricemia was shown to mediate mitochondrial calcium overload and eventually cause endothelial dysfunction through mitochondrial Na^+^/Ca^2+^ exchange, which increases the production of ROS [47]. The mechanism of UA-induced endothelial dysfunction is closely associated with reduced mitochondria mass and ATP production [48]. Although mitochondria in TECs may undergo substantial damage under oxidative stress, glutathione (GSH) treatment shows an effective resistance as an antioxidant [46]. Another important source of ROS is NADPH oxidase. UA has been found to stimulate the synthesis of ROS by NADPH oxidase in various cells, such as adipocytes, vascular smooth muscle cells, and vascular endothelial cells [49]. Using stable isotope labeling with amino acids in cell culture and liquid chromatography-tandem mass spectrometry (LC-MS/MS) to analyze differentially expressed proteins and the functional status of UA-stimulated human umbilical vein endothelial cells (HUVECs), Zhang et al. [50] found a significant relationship between hyperuricemia-induced endothelial dysfunction and aldose reductase- (AR-) mediated oxidative stress. Meanwhile, it has been reported that hyperuricemia induced endothelial dysfunction via regulation of AR, while inhibition of AR or degradation of ROS could restore endothelial function [51]. Similarly, antioxidant therapies, such as tempol and reduced GSH, may be beneficial for the recovery of endothelial function [45, 46]. Taken together, hyperuricemia-mediated oxidative stress directly damages the kidney, thus being a biotherapeutic target for UA-induced renal damage.

### 3.3. Hyperuricemia-Induced Endothelial Dysfunction

The renin-angiotensin system (RAS) mainly regulates the cardiovascular function and maintains the body fluid balance in cooperation with other compensatory mechanisms. Evidence from animal and patient studies showed that UA-mediated RAS activation is closely related to diabetic complications, such as cardiovascular and kidney diseases [52]. Upon UA stimulation, the expression of angiotensinogen, angiotensin-converting enzyme (ACE), and angiotensin II receptors was notably upregulated *in vitro*, resulting in the inhibition of proliferation and promotion of senescence, inflammation, and apoptosis of endothelial cells [53]. Moreover, UA-induced senescence and apoptosis of HUVECs were blocked by enalaprilat (an ACE inhibitor) or telmisartan (an angiotensin II receptor antagonist). These findings indicated that RAS activation was a novel mechanism of UA-induced endothelial dysfunction [40].

Endothelial cells secrete various vasoactive substances to regulate the relaxation and contraction of blood vessels, including the potent vasoconstrictor endothelin 1 (ET-1) and the effective vasodilator nitric oxide (NO) [54]. An imbalance between ET-1 and NO drives endothelial dysfunction, which plays an essential role in the pathophysiology of cardiovascular and renal diseases. Under physiological conditions, ET-1 stimulates the synthesis of NO by endothelial cells, while NO exerts a negative feedback effect on ET-1 [55]. Overexpression of ET-1 may elevate blood pressure and cause vascular and kidney injuries, such as reduced renal artery flow and small artery stiffening [56]. Accumulating evidence indicates that UA impacts endothelial function through downregulation of NO production and endothelial nitric oxide synthase (eNOS) activity, which subsequently decreases NO bioavailability. UA was shown to affect the activity of eNOS and production of NO in a dose- and time-dependent manner [57]. L-arginine is the substrate of eNOS and is converted to NO in mammalian endothelial cells [58]. However, it was reported that UA could not only stimulate arginase, an enzyme degrading L-arginine, but also enhance the affinity of L-arginine to arginase, which reduced the availability of the substrate for NO synthesis [58]. In addition, recent studies have demonstrated that UA markedly reduced the binding between eNOS and calmodulin (CaM), an eNOS activator, in both HUVECs and bovine aortic endothelial cells [57, 59]. Zhang et al. [60] suggested that high-level UA can induce endothelial dysfunction through miR-155-mediated eNOS suppression. In addition, UA regulated the PKC/eNOS pathway and endoplasmic reticulum (ER) stress, leading to endothelial dysfunction and apoptosis in HUVECs [57]. Although it was clearly demonstrated that UA inhibited eNOS activity and the interaction between eNOS and CaM, it did not influence the expression of eNOS and the intracellular amount of CaM [59]. The reason for the latter is not fully understood and may be related to posttranslational modifications and the activation of eNOS.

UA induces endothelial dysfunction via various pathways, while targeted therapy may ameliorate the endothelial dysfunction and alleviate kidney damage. It has been reported that iptakalim, an ATP-sensitive potassium channel opener, could improve endothelial dysfunction and defend against hypertension and hyperuricemia [60]. Additionally, XO inhibitors, such as allopurinol and febuxostat, exhibited protective effects on endothelial dysfunction in clinical therapy and animal models [24, 61, 62].

### 3.4. Hyperuricemia-Induced Renal Fibrosis

Renal fibrosis, characterized by glomerulosclerosis and tubulointerstitial fibrosis, is a common pathological process in all patients with CKD, leading to the loss of effective nephrons and a progressive decline in renal function, resulting in ESRD [63]. A clinical study of 1,700 biopsy-confirmed patients demonstrated that patients with high levels of plasma UA displayed not only more serious clinical renal dysfunction but also more severe renal pathology, particularly segmental glomerulosclerosis and tubular atrophy/interstitial fibrosis [64]. Recently, a line of evidence has indicated that hyperuricemia may directly cause glomerulosclerosis and tubulointerstitial fibrosis.

#### 3.4.1. Hyperuricemia-Induced Glomerulosclerosis

Mild hyperuricemia causes renal arteriolosclerosis and glomerular hypertension and disrupts renal autoregulation, ultimately resulting in glomerulosclerosis [65]. Recently, the deleterious effects of hyperuricemia on glomerular intrinsic cells were explored, which may uncover the potential mechanisms of hyperuricemia-induced glomerulosclerosis.

As mentioned in the previous section, multiple pathways are involved in hyperuricemia-induced endothelial cell injury; thus, mild hyperuricemia may impair glomerular endothelial cells via similar mechanisms. In addition, similar to epithelial-to-mesenchymal transition (EMT), endothelial-to-mesenchymal transition (EndoMT) greatly contributes to the activation of fibroblasts and myofibroblasts and subsequent renal fibrosis [66]. EndoMT contributed to renal fibrosis in three mouse models of CKD, including unilateral ureteral obstructive nephropathy, streptozotocin-induced DN, and Alport kidney disease models [67–69]. These studies suggested that endothelial-origin myofibroblasts possibly contribute to the progression of glomerulosclerosis [68, 70]. Recent research has demonstrated that UA induced the phenotype transition in HUVECs via induction of oxidative stress and glycocalyx shedding [71]. However, direct evidence is still lacking for the contribution of UA-induced EndoMT to glomerulosclerosis. Therefore, further studies are needed to identify the role of UA-induced EndoMT in glomerulosclerosis by utilizing a conditionally immortalized human glomerular cell line [72] *in vitro* and an endothelial lineage-traceable mouse line *in vivo* [68].

Abnormal proliferation of glomerular mesangial cells (MCs) and overproduction of extracellular matrix (ECM) contribute to glomerulosclerosis. UA-mediated activation of the COX-2/mPGES-1/PGE2 inflammatory cascade not only has a direct proinflammatory effect but also induced the proliferation of MCs [73, 74]. Moreover, UA time-dependently induced MC proliferation through the NADPH/ROS/extracellular signal-regulated kinase (ERK)1/2 signaling pathway [75]. Albertoni et al. [76] proved that soluble UA stimulated the proliferation and contraction of human MCs via an angiotensin II-dependent mechanism and the production of ET-1 *in vivo*, which might have a long-term effect on glomerular function. In addition, soluble UA induced ER stress by upregulating the expression of *α*-smooth muscle actin (*α*-SMA), fibronectin (FN), and transforming growth factor-*β* 1 (TGF-*β*1) in a time- and concentration-dependent manner, which resulted in a phenotypic change in rat glomerular MCs [77]. Thus, UA-induced proliferation of glomerular MCs and production of extracellular matrix may lead to glomerular hypertrophy and sclerosis. These novel mechanisms suggest some potential targets for the treatment of hyperuricemia-induced glomerulosclerosis.

Notably, hyperuricemia also influences the function of glomerular podocytes, a key player in maintaining the glomerular filtration barrier, leading to albuminuria. Electron microscopy of kidney biopsies from patients with gout revealed varying degrees of podocyte proliferation and damage [78]. Signs of significant albuminuria were found in hyperuricemic model rats, accompanied by upregulation of desmin, a podocyte injury marker, and downregulation of podocin, a key component of the podocyte slit diaphragm [79, 80].

#### 3.4.2. Hyperuricemia-Induced Renal Interstitial Fibrosis

Myofibroblasts act as collagen-producing cells in various pathologies, including renal interstitial fibrosis. During renal interstitial fibrosis, half of the myofibroblasts are derived from renal resident fibroblasts [81]. Under the stimulation of cytokines and growth factors, fibroblasts undergo activation and proliferation, achieve myofibroblast phenotype, and synthesize ECMs, including structural scaffolds, fibronectin, and various types of collagens [82]. UA promotes the renal fibroblast–myofibroblast transition mainly through the activation of the TGF-*β*/Smad3, epidermal growth factor receptor (EGFR), and ERK1/2 pathways [83, 84]. However, after treatment with 3-deazaneplanocin A, a selective inhibitor of the enhancer of zeste homolog 2, the above-activated pathways were inhibited, and the proliferation of renal fibroblasts was suppressed, alleviating renal interstitial fibrosis [83].

EMT is a physiological or pathophysiological process, leading to the phenotype transformation of renal tubular cells, which lose their epithelial phenotype and acquire that of mesenchymal cells [63, 85, 86]. It has been reported that almost one-third of myofibroblasts originate from EMT rather than from preexisting local fibroblasts [87]. EMT plays a primary role in the accumulation of myofibroblasts and the resulting production of ECM, which are the key steps in the progression of renal interstitial fibrosis [86, 88]. EMT is often accompanied by a decreased expression of the epithelial cell marker E-cadherin via upregulation of Snail and Slug and an increased expression of mesenchymal cell markers, such as *α*-SMA, vimentin, fibronectin, and smooth muscle 22 (SM22) [86]. Substantial evidence indicates that the TGF-*β*/Smad3 pathway plays a dominant role in the progression of EMT [84, 89, 90]. It has also been shown that UA activates the TGF-*β*/Smad3 signaling pathway in type 2 DN, promoting EMT and profibrogenic progression [18]. Setyaningsih et al. [91] reported that hyperuricemia induced EMT and kidney tubular injury in mice via regulation of the Wnt5a/Ror2 signaling pathway. Zhou et al. [63] also found that UA caused EMT through the stimulation of the toll-like receptor 4 (TLR4)/nuclear factor-kappa B (NF-*κ*B) pathway. Furthermore, hyperuricemia may induce EMT through the PI3K/Akt signaling pathway [92]. EMT has already been deemed a new therapeutic target due to its reversibility [91, 93, 94]. Studies have demonstrated that UA-induced EMT could be inhibited by probenecid, an organic anion transport inhibitor [66]. In addition, in a hyperuricemia nephropathy rat model, a traditional Chinese medicine decreased the UA level and relieved renal interstitial fibrosis via inhibition of the EMT process [95]. Tao et al. [96] demonstrated that UA-induced EMT was prevented after blocking ERK1/2 with the specific inhibitor U0126.

Matrix metalloproteinases (MMPs) are zinc-dependent endopeptidases involved in the degradation of extracellular and basement membranes [97]. It was shown that MMPs could promote EMT and establish a profibrotic environment, which may contribute to renal interstitial fibrosis [98–100]. Reports have shown that MMP2 and MMP9 were significantly activated in renal tissue of hyperuricemic rats [96]. Moreover, inhibition of the NF-*κ*B/MMP9 signaling pathway by chloride channel 5 (CIC-5) overexpression suppressed TGF-*β*1-induced EMT [101]. ER stress is a cellular physiological or pathological response to the accumulation of misfolded and mismatched proteins in ER [102]. ER stress is closely associated with renal fibrosis [102, 103]. Recently, He et al. [104] found that a marker of ER stress (RTN1A) was markedly upregulated in hyperuricemic nephropathy; however, febuxostat suppressed ER stress, thereby improving kidney injury and interstitial fibrosis [105]. Thus, MMPs and ER stress may be additional hallmarks and therapeutic targets for hyperuricemia-induced renal interstitial fibrosis.

### 3.5. Hyperuricemia-Induced Renal Inflammation

During necrosis, the dying cell releases amount of danger signals, such as ATP, high-mobility group box protein 1 (HMGB1), heat shock proteins, and UA, to activate immune response. UA may crystallize into MSU crystal in the extracellular fluid and can be recognized by pattern recognition receptors (e.g., TLRs) expressed on antigen-presenting cells (APCs, such as macrophages and TECs) as one of the danger-associated molecular patterns (DAMPs), which ultimately activates immune and inflammatory responses. Notably, hyperuricemia may induce renal inflammation via crystal-dependent and crystal-independent pathways [106].

#### 3.5.1. MSU Crystal-Induced Renal Inflammation

It is generally accepted that hyperuricemia induces renal inflammation in a crystal-dependent manner. Macrophages are considered key mediators and have been studied most in MSU crystal-induced renal inflammation. MSU crystals deposited in the tubular lumen or interstitial space can be recognized and engulfed into renal resident or infiltrated macrophages [63, 107]. Upon stimulation with MSU crystal, the production of chemokines, such as CXCL-12, which induce directional chemotaxis in nearby inflammatory cells, was significantly enhanced in tubular cells possibly leading to the accelerated renal recruitment of macrophages [108]. Moreover, MSU crystals strongly activated human primary macrophages to secrete the lysosomal protease cathepsin, proinflammatory cytokines, such as interleukin- (IL-) 1*β*, IL-18, and interferon through the Src/Pyk2/PI3K signaling pathway [109]. Nod-like receptor pyrin domain-containing protein 3 (NLRP3), an important member of NLRs, senses danger signals, including pathogen-associated molecular patterns (PAMPs) and DAMPs, in the cytosol and activates sterile inflammation [108, 110]. NLRP3 then assembles the functional NLRP3 inflammasome, which subsequently leads to the transformation of immature pro-IL-1*β* and pro-IL-18 into mature, bioactive IL-1*β* and IL-18, respectively, ultimately activating the entire cascade and amplifying downstream inflammatory signals [111]. Through endocytosis into macrophages, lysosomes capture MSU crystal for degradation; however, MSU crystal cannot be degraded but instead ruptures the lysosomal membrane and releases lysosomal cathepsins into the cytoplasm, leading to the activation of the inflammasome [33]. Active caspase-1 may cleave gasdermin D (GSDMD) into GSDMD-N, triggering cell pyroptosis [112, 113]. Interestingly, MSU crystal-induced macrophages not only secrete proinflammatory cytokines at the inflammatory activation stage but also produce anti-inflammatory cytokines during the resolution phase of inflammation. In particular, it was reported that MSU crystals promoted macrophages to secrete TGF-*β*1 through mediation of the metastatic tumor antigen 1 (MTA1)/transglutaminase 2 (TG2) signaling pathway, which contributed to self-limitation of inflammation [114]. TGF-*β*1 acts as a strong profibrotic cytokine, and aberrant TGF-*β*1 derived from MSU crystal-induced macrophages may promote renal fibrosis. Besides macrophages, infiltrated T cells not only could phagocytize MSU crystals but also could be directly activated and stimulated to proliferate by MSU crystals in the absence of APC [115].

Injured TECs also produce a lot of cytokines and chemokines to promote renal inflammation. Urate crystals can adhere to renal TECs through hydrogen bonding and hydrophobic interactions to induce TEC injury [116]. In addition, damaged TECs rapidly secrete migration inhibitory factor (MIF), a mediator of delayed-type hypersensitivity, to recruit macrophages and other immune cells [117]. MSU crystal also induces NLRP3 inflammasome activation in TECs triggered by lysosomal rupture. Released lysosomal cathepsins can initiate RIP3/MLKL-dependent necroptosis, which was confirmed by a study that showed that RIP3 deficiency attenuated hyperuricemia-caused tubular injury and renal inflammation in mice [118]. Necroptosis of TECs leads to the release of danger signals, further promoting renal inflammatory response in a positive feedback loop.

#### 3.5.2. Soluble UA-Induced Renal Inflammation

Recent studies suggest that soluble UA may also have proinflammatory effects, independent of crystal formation. Braga et al. [119] found that soluble UA could also stimulate the activation of the NLRP3 inflammasome and the synthesis of IL-1*β in vivo* and *in vitro*. Moreover, soluble UA activated NLRP3 inflammasome to secrete IL-1*β* in macrophages and stimulated the release of CXCL12 and HMGB1 in TECs, while interaction between macrophages and TECs promoting the progression of DN [108]. Soluble UA significantly enhanced NLRP3, tumor necrosis factor- (TNF-) *α* as well as IL-1*β* in TECs, while AMP-activated protein kinase (AMPK) exerted a protective effect on UA-induced inflammatory response [120]. In the absence of lysosomal ruptures, mitochondria-derived ROS may mediate soluble UA-activated NLRP3 inflammasome [119]. In a rat model, hyperuricemia induced renal inflammation and promoted the progression of renal disease via a monocyte chemoattractant protein-1- (MCP-1-) related mechanism [121]. In cultured TECs (NRK-52E) and a hyperuricemia mouse model, UA induced the infiltration of inflammatory cells (T cells and macrophages) in tubular interstitial spaces and upregulated the production of the inflammatory cytokine tumor necrosis factor-*α* (TNF-*α*) and MCP-1 and regulated upon activation normal T cell expressed and secreted factor (RANTES) expression via the NF-*κ*B signaling pathway [63]. In another rat model, soluble UA stimulated the production of MCP-1 in vascular smooth muscle cells (VSMCs), subsequently promoting the infiltration of inflammatory cells in kidney, causing profound renal vasoconstriction and chronic renal injury [122–124]. Soluble UA was also shown to directly stimulate proinflammatory cytokine production in human peripheral blood mononuclear cells (PBMCs) through breaking the IL-1*β*/IL-1 receptor antagonist (IL-1Ra) balance [125]. Plasma UA was found to induce endothelial dysfunction and inflammation in renal allograft recipients, which might lead to chronic renal allograft damage [126]. Therefore, the reduction in UA levels may bring many benefits. For example, after urate-lowering therapy (ULT) with benzbromarone in healthy volunteers for two weeks, the serum inflammatory cytokine IL-18 was significantly decreased [127]. Moreover, lowering plasma UA levels markedly decreased renal damage, the expression of MCP-1, and the macrophage M1/M2 ratio in a hyperuricemic mouse model [128].

High levels of UA significantly upregulate the expression of HMGB1 through activation of TLR4 and the MEK/ERK pathway [129]. HMGB1 amplifies inflammatory responses via various pathways, including the promotion of mononuclear cells to secrete proinflammatory cytokines, such as IL-1*β* and TNF-*α*, the expression of adhesion molecules, and inflammatory cell infiltration [130]. In particular, HMGB1 promotes its own release from endothelial cells by a positive-feedback mechanism. After binding to the receptor of advanced glycation end products (RAGE), UA-induced HMGB1 activates the NF-*κ*B signaling pathway and promotes the production of cytokines, such as TNF-*α* and IL-6, leading to oxidative stress and inflammatory responses [131].

Recent evidence has demonstrated another inflammatory mechanism of renal damage, which involves hyperuricemia-induced Na^+^/K^+^-ATPase (NKA) degradation in lysosomes, whereas AMPK was shown to alleviate NKA downstream inflammation and maintain renal function. AMPK activators, such as metformin and 5-aminoimidazole-4-carboxamide-1-*β*-D-ribofuranoside (AICAR), significantly relieved hyperuricemia-induced renal damage and NKA signaling impairment in a rat model, although these compounds did not lower the serum UA levels in rats [132]. Recent studies have confirmed that autophagy also plays a functional role in hyperuricemia-induced inflammation [33, 133, 134]. Activation of autophagy may limit inflammasome activity induced by hyperuricemia through targeting ubiquitinated inflammasomes for degradation [135] and decreasing the production of ROS [136] and downstream inflammatory responses [137]. However, Bao et al. [138] demonstrated that the inhibition of autophagy with 3-methyladenine (3-MA) not only delayed the progression of renal fibrosis but also suppressed the infiltration of immune cells and the secretion of various inflammatory cytokines. More evidence is needed to explore the protective or deleterious role of autophagy in hyperuricemia-induced renal inflammation.

## 4. Perspective and Conclusion

Currently, the standard treatment for patients with hyperuricemia is ULT, mainly including the XO inhibitors allopurinol and febuxostat, the UA reabsorption inhibitor benzbromarone and urate oxidase (rasburicase). In 2012, the American College of Rheumatology recommended using either allopurinol or febuxostat for first-line ULT [139]. In contrast to XO inhibitors, rasburicase lowers hyperuricemia quickly but does not induce the accumulation of xanthine, which is usually used in the prevention and treatment of tumor lysis symptoms. XO inhibitors may exert renoprotective effects beyond lowering UA. XO produces ROS, and inhibition of XO by allopurinol or febuxostat attenuates ROS-mediated kidney injury [140–142]. Some drugs targeting URAT1, such as SHR4640 and RDEA3170, are still under clinical trials, which may raise new hope for the treatment of hyperuricemia.

Apart from classical ULT drugs, losartan, as an angiotensin II receptor blocker, has been proven to reduce serum UA via inhibiting UA reabsorption mediated by URAT1 in TECs [143, 144]. Sodium-glucose cotransporter 2 inhibitors, which are approved antidiabetic drugs, promote UA excretion by suppressing UA reabsorption via commandeering UA transporter GLUT9 [145, 146].

Traditional medicines were also reported to reduce the level of serum UA and attenuate hyperuricemia-induced kidney injury. Extracts from *Urtica hyperborea* Jacq. Ex Wedd. significantly reduced the renal expression of URAT1 and increased that of OAT1, thereby lowering the serum UA level and improving renal injury [147]. Epigallocatechin gallate exerted hypouricemic effects by suppressing XO activity and GLUT9 expression and promoting OAT1 expression *in vivo* and *in vitro* [148]. Tu-Teng-Cao extract remarkably decreased the concentration of serum UA in potassium oxonate-induced hyperuricemia rats [149].

However, there is a long way to translate the identified novel mechanisms of hyperuricemia-induced renal injury based on experimental studies into clinical applications. Research methods used for hyperuricemia-induced renal injury have certain limitations. For instance, most of the *in vivo* and *in vitro* studies related to hyperuricemia-induced renal injury involve simple experimental models; however, clinical patients are more complex, and most of them either have a different underlying disease or various accompanying complications. Hence, complex models, such as models of CKD accompanied by hyperuricemia or hyperuricemia models with an AKI attack, should be considered because they might be more in line with the actual situation in clinical patients. Meanwhile, with the rapid development of modern computers and gene-related technologies, more emphasis should be placed on novel medical approaches, such as gene and metabolic pathway analyses, as well as on the combination of the modern information technology and clinical cases, which may become a new direction in the research of hyperuricemia-induced renal injury.

**Figure 1:**
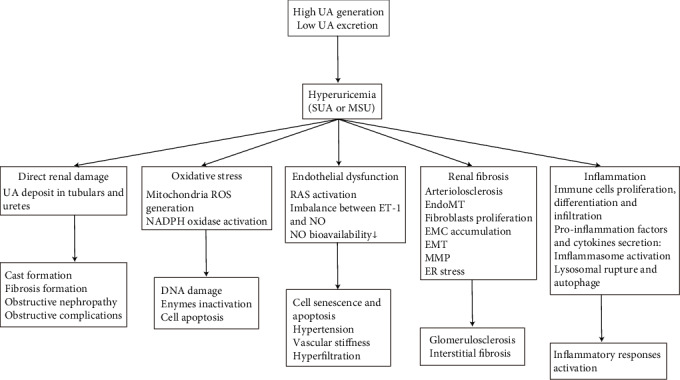
Mechanisms of hyperuricemia-induced renal injury. UA: uric acid; SUA: soluble uric acid; MSU: monosodium urate; ROS: reactive oxygen species; RAS; renin-angiotensin system; ET-1: endothelin 1; NO: nitric oxide; EndoMT: endothelial-to-mesenchymal transition; EMT: epithelial-to-mesenchymal transition; MMP: matrix metalloproteinase; ER stress: endoplasmic reticulum stress.

In conclusion, with a progressively higher incidence, hyperuricemia not only increases the risks but also affects the prognosis of renal diseases. The mechanisms of hyperuricemia-induced renal injury mainly include oxidative stress, endothelial dysfunction, renal fibrosis, and inflammation (Figure 1). However, the whole mechanisms of hyperuricemia-caused renal injury are complex and not fully understood, thus requiring further research. The novel underlying mechanisms may contribute to the development of clinical therapies with the potential to improve the treatment of hyperuricemia and hyperuricemia-caused renal injury.

## Abbreviations

ACE: Angiotensin-converting enzyme
AICAR: 5-aminoimidazole-4-carboxamide-1-*β*-D-ribofuranoside
AKI: Acute kidney injury
AMPK: AMP-activated protein kinase
APC: Antigen-presenting cell
AR: Aldose reductase
BCRP/ABCG2: Breast cancer resistance protein
CaM: Calmodulin
CIC-5: Chloride channel 5
CKD: Chronic kidney disease
DAMP: Danger-associated molecular pattern
DN: Diabetic nephropathy
ECM: Extracellular matrix
eGFR: Estimated glomerular filtration rate
EGFR: Epidermal growth factor receptor
EMT: Epithelial-to-mesenchymal transition
EndoMT: Endothelial-to-mesenchymal transition
eNOS: Endothelial nitric oxide synthase
ER: Endoplasmic reticulum
ERK: Extracellular signal-regulated kinase
ESRD: End-stage renal disease
ET-1: Endothelin 1
FN: Fibronectin
GLUT9: Glucose transporter 9
GSH: Glutathione
GSDMD: Gasdermin D
HMGB1: High-mobility group box protein 1
HUVEC: Human umbilical vein endothelial cell
IL: Interleukin
LC-MS/MS: Liquid chromatography-tandem mass spectrometry
3-MA: 3-methyladenine
MCs: Mesangial cells
MCP-1: Monocyte chemoattractant protein-1
MMP: Matrix metalloproteinase
MRP4/ABCC4: Multidrug resistance protein 4
MSU: Monosodium urate
MTA1: Metastatic tumor antigen 1
NF-*κ*B: Nuclear factor-kappa B
NKA: Na^+^/K^+^-ATPase
NLRP3: Nod-like receptor pyrin domain-containing protein 3
NO: Nitric oxide
OAT: Organic anion transporter
PAMP: Pathogen-associated molecular pattern
PBMC: Peripheral blood mononuclear cells
RAS: Renin–angiotensin system
ROS: Reactive oxygen species
*α*-SMA: *α*-Smooth muscle actin
SM22: Smooth muscle 22
TECs: Tubule epithelial cells
TG2: Transglutaminase 2
TGF-*β*: Transforming growth factor-*β*
TLR: Toll-like receptor
TNF-*α*: Tumor necrosis factor-*α*
UA: Uric acid
ULT: Urate-lowering therapy
URAT1: Urate anion transporter 1
VSMC: Vascular smooth muscle cell
XO: Xanthine oxidase.
